# A cluster randomised controlled trial of the community effectiveness of two interventions in rural Malawi to improve health care and to reduce maternal, newborn and infant mortality

**DOI:** 10.1186/1745-6215-11-88

**Published:** 2010-09-17

**Authors:** Sonia Lewycka, Charles Mwansambo, Peter Kazembe, Tambosi Phiri, Andrew Mganga, Mikey Rosato, Hilda Chapota, Florida Malamba, Stefania Vergnano, Marie-Louise Newell, David Osrin, Anthony Costello

**Affiliations:** 1 Centre for International Health and Development, UCL Institute of Child Health, 30 Guilford St, WC1N 1EH, London, UK; 2 Kamuzu Central Hospital, Ministry of Health, Lilongwe, Malawi; 3 Baylor College of Medicine Children's Foundation, Private Bag B-397, Lilongwe, Malawi; 4 MaiMwana Project, PO Box 2, Mchinji, Malawi; 5 St George's Hospital NHS Trust, Paediatric Infectious Diseases, Blackshaw Road, London SW17 0BY, UK; 6 Africa Centre for Health and Population Studies, University of KwaZulu-Natal, South Africa

## Abstract

**Background:**

The UN Millennium Development Goals call for substantial reductions in maternal and child mortality, to be achieved through reductions in morbidity and mortality during pregnancy, delivery, postpartum and early childhood. The MaiMwana Project aims to test community-based interventions that tackle maternal and child health problems through increasing awareness and local action.

**Methods/Design:**

This study uses a two-by-two factorial cluster-randomised controlled trial design to test the impact of two interventions. The impact of a community mobilisation intervention run through women's groups, on home care, health care-seeking behaviours and maternal and infant mortality, will be tested. The impact of a volunteer-led infant feeding and care support intervention, on rates of exclusive breastfeeding, uptake of HIV-prevention services and infant mortality, will also be tested. The women's group intervention will employ local female facilitators to guide women's groups through a four-phase cycle of problem identification and prioritisation, strategy identification, implementation and evaluation. Meetings will be held monthly at village level. The infant feeding intervention will select local volunteers to provide advice and support for breastfeeding, birth preparedness, newborn care and immunisation. They will visit pregnant and new mothers in their homes five times during and after pregnancy.

The unit of intervention allocation will be clusters of rural villages of 2500-4000 population. 48 clusters have been defined and randomly allocated to either women's groups only, infant feeding support only, both interventions, or no intervention. Study villages are surrounded by 'buffer areas' of non-study villages to reduce contamination between intervention and control areas. Outcome indicators will be measured through a demographic surveillance system. Primary outcomes will be maternal, infant, neonatal and perinatal mortality for the women's group intervention, and exclusive breastfeeding rates and infant mortality for the infant feeding intervention.

Structured interviews will be conducted with mothers one-month and six-months after birth to collect detailed quantitative data on care practices and health-care-seeking. Further qualitative, quantitative and economic data will be collected for process and economic evaluations.

**Trial registration:**

ISRCTN06477126

## Background and rationale

There has been less than satisfactory progress, especially in sub-Saharan Africa, towards the child and maternal mortality targets of Millennium Development Goals (MDGs) 4 and 5. The Countdown group reported that most of 68 target countries had made little or no progress towards the child survival target, and that 12 countries in sub-Saharan Africa had actually seen reversals in child survival rates [1]. The neonatal period has been especially resistant to change and, as mortality in later infancy has fallen, globally the proportion of under-five deaths occurring in the newborn period is now close to 40%.

Our study will evaluate two community-based interventions aimed at reducing newborn and infant mortality rates through either mobilisation of women's groups or home visits by village women volunteers focusing on maternal and infant care and feeding practices. Several previous trials have evaluated similar interventions in south Asia, but none have examined these approaches in sub-Saharan Africa, in settings where HIV, malaria, maternal and newborn mortality are common.

Our study uses a cluster-randomised and controlled design with buffer zones to reduce the risk of contamination. We hope it will provide policymakers with new and evidence-based options for strategies to reduce newborn and child mortality.

### The importance of maternal, infant and neonatal health in Malawi

The maternal mortality ratio in Malawi has been reported as 984 per 100 000 [2] - one of the highest in the world. Neonatal mortality accounts for about one third of infant mortality; the Demographic and Health Survey report for 2004 indicates an infant mortality ratio (IMR) of 76 per 1000 and a neonatal mortality ratio (NMR) of 27 per 1000 [2]. For women who seek contact with health services, the quality of care they receive may be compromised due to low public health expenditure, high turnover of service providers, lack of drugs and supplies in many facilities, and lack of ownership of health programmes by local communities [3]. Further, in rural Malawi more than half of deliveries occur at home, and these home deliveries may be more likely to result in adverse maternal or perinatal outcomes.

### Essential newborn care and safe motherhood

Essential newborn care (ENC) is based on the simple principles of basic resuscitation, avoidance of hypothermia, improvements in perinatal hygiene, early breastfeeding and protecting maternal-infant bonding. It also promotes antenatal care, delivery at health facilities and the treatment or referral of high risk or symptomatic infants. ENC forms an integral part of Safe Motherhood Programmes (SMP) and the WHO Integrated Management of Childhood Illness (IMCI) strategy. Currently, WHO and UNICEF aim to improve newborn care through the 'integrated management of pregnancy and childbirth' (IMPAC) package as part of SMP, and post-perinatal mortality through the IMCI strategy. Both strategies recognise that improvement in health status in poor communities is strongly related to family caretaking practices.

### Exclusive breastfeeding

Exclusive breastfeeding is recommended worldwide, but not always practised. Where supplementary or replacement foods are given to young infants, there is a risk of compromising infant nutrition and of introducing infection. Women with HIV can reduce the risk of vertical transmission by choosing not to breastfeed [4,5], but in rural Africa this is not a viable option. Formula feeds are expensive and local water supplies likely to be unclean, and nutritious replacement foods may be unaffordable. There is clear evidence that increased duration and exclusivity of breastfeeding is associated with decreased diarrhoea incidence and better infant survival [6-8]. Current WHO guidelines for the HIV uninfected mother or of unknown status recommend exclusive breastfeeding of their infants for the first six months of life, introducing appropriate complementary foods thereafter, and continue breastfeeding for the first 12 months of life. Breastfeeding should then only stop once a nutritionally adequate and safe diet without breast-milk can be provided [5].

According to the Malawi DHS [2], only 28% of children 4-5 months of age are exclusively breastfed, and median duration of exclusive breastfeeding is 2.5 months. Infants may be given water or a watery porridge as early as one week of age. This may be because the mother is perceived to not be producing enough milk, or the baby is believed to be crying excessively. Traditional remedies and home-made gripe-water may also be given to strengthen the baby and protect from illness ([9]; and MaiMwana baseline research). Early introduction of complementary feeds can increase the risk of gastrointestinal infection and growth faltering [10,11].

### HIV and maternal and child health

HIV is an important issue in any health intervention where prevalence is high. It has a direct impact on the health of those infected with the virus, and indirect impact through the larger societal effects of HIV-related illness in people of working age, such as key health workers, and an increased burden on the family of caring for sick people and orphans. Children born to mothers with HIV are at increased risk of dying, even when they themselves are uninfected [12]. However, in the absence of any intervention, 30-45% of children born to mothers with HIV will become infected through in-utero, peripartum or breast milk exposure, and, without treatment, 35% of these will die before they are one year old [13]. Initiatives such as the Global Fund have helped to make services for prevention of mother-to-child transmission of HIV (PMTCT) more widely available in Malawi, including HIV testing during antenatal care, free treatment for HIV-positive mothers and babies, and breastfeeding advice. Malawi's estimated HIV prevalence among women between 15 and 49 years is approximately 13.3% [2], and there is a need for appropriate interventions to reduce the risk of MTCT. Single dose Nevirapine was adopted as the minimum prophylaxis regimen in national policy - one dose for mothers at onset of labour and one dose for infants immediately after birth. This has been shown to reduce MTCT by 40% in a trial setting [14]. The Ministry of Health has now introduced a combination antiretroviral prophylaxis regimen at facilities with the capacity to offer this.

### Why use community-based interventions?

The rationale for using community-based interventions is based on the fact that many maternal and neonatal deaths occur at home, and could potentially be avoided by changes in antenatal and newborn care practice and better understanding of health problems. Stimulating uptake and improving coverage of health services must also begin at community level. Consolidation of the links between primary health care services and their users is essential, as described in the Alma-Ata Declaration [15]. This involves both improving the quality of the services and creating demand and awareness in communities. Reasons for under-use of existing services are complex, and in order to increase uptake, not only must physical barriers to access be removed, but issues of service quality and community perceptions of service providers must be addressed.

### Why promote participation in health care?

Community-based health interventions do not always involve the community in decision-making. As there are different perceptions of priorities between officials and communities, communities may feel their needs are not being met, and powerful groups may capture resources [16-18]. Groups most consistently excluded from decision-making have been women and children. Involving groups who have traditionally had little influence in decision-making is important because community members have experience and insight that can lead to more appropriate decisions, increased community commitment to planned work ensures its sustainability, the community gains some power and control over any planned work, and there may be more resources available for the work as local materials and manpower can be used.

Furthermore, simply providing people with information about health risks is not always enough to change behaviour. Health is indirectly but powerfully affected by the social environment in which personal behaviours are embedded [19]. In Nepal, providing direct one-to-one basic information on infant care and family planning did not result in significant changes in behaviour [20]. Similarly, a review of HIV prevention projects found only one in four participants on average can be expected to change behaviour through individually focused behavioural interventions [21]. More success has been achieved through health alliances or partnerships, and the stronger the representation by the community, and the greater the community involvement in the practical activities of the health promotion, the greater the impact and the more sustainable the gains. A social change approach takes the focus away from individuals and encourages community responsibility for action. Community-based programmes can "help to provide enabling conditions for the renegotiation of [behaviour] at the collective level, rather than attempting to persuade people to make an individual decision to change their behaviour by simply providing them with information about health risks" [22,23].

### Model strategies for women's group community mobilisation interventions

The Warmi Project - introduced in a rural area of Bolivia with little health infrastructure and widespread poverty - was the first published account of a community participatory intervention to improve perinatal care [24-26]. It employed participatory planning methods and community action cycles focused on mother and infant care. Groups of women worked together to identify and prioritise key maternal and neonatal health problems, then developed local strategies to address them. Within three years, the Warmi Project had achieved a substantial decrease in the perinatal mortality rate, from 117 to 44 per thousand births. However, it is difficult to draw firm conclusions because the study lacked a control group and had relatively low statistical power.

The MIRA Makwanpur study, Nepal - the main operational model for this project - was a cluster-randomised controlled trial of a community-based participatory intervention to improve the health of pregnant mothers and newborn infants [27]. MIRA used community action cycles run by women's groups, based on the Warmi model, to bring about improvements in perinatal health outcomes. Over a two-year period, neonatal mortality was reduced by 30% in intervention clusters compared with controls. The intervention used trained local facilitators to mobilise the groups.

Another recent study, from Jharkhand and Orissa in India, showed similar impact of women's groups. Neonatal mortality was reduced by 45% in intervention areas in the second and third years (OR 0.55 95% CI 0.46-0.66) [28]. However, such major changes were not seen in a study from Bangladesh, which may have been due to limited coverage of community activities [29].

### Model strategies for infant feeding and care counselling interventions

Three cluster-randomised controlled trials conducted in Mexico, Bangladesh and India have shown significant impacts of peer-led counselling on exclusive breastfeeding rates and reduced infant illness. In Mexico City, follow-up at 3 months after birth found that 67% and 50% of the intensive and less intensive counselling intervention groups respectively were still exclusively breastfeeding, compared to 12% of the control group [6], and both intervention groups had less diarrhoea than control infants. In Dhaka, Bangladesh, follow-up at 3 months and 5 months after birth found 83% and 70% respectively of women in the intervention areas exclusively breastfeeding, compared with 18% and 6% in control areas [30]. In these two interventions the counsellors were paid employees of the projects, but in Haryana State, India, they used volunteer counsellors, and also included monthly community meetings and opportunistic visits by community-based health workers and traditional birth attendants. At 3 months after birth, 79% of infants in the intervention areas were exclusively breastfed, compared with 48% in control areas. The difference was still significant at 6 months (after which complementary feeds were introduced), with 42% of women in intervention areas exclusively breastfeeding, compared with 4% in control areas [8].

The best work on infant feeding in Africa has been done by LINKAGES, which works with Ministries of Health and other partners to promote breastfeeding by enlisting the support of families and communities, and by building a critical mass of breastfeeding advocates for community programmes. LINKAGES programmes in Africa have already had substantial impacts judged by before-and-after analyses [31]. In Madagascar, exclusive breastfeeding rates increased from 46% to 68% and in Ghana rates increased from 31% to 68%. Whilst the LINKAGES work provides interesting models for implementation, their community cost-effectiveness on a larger scale remains uncertain, as the evaluation of the intervention is not based on an experimental, randomised design, and the economic evaluation has not yet been reported.

### Justification for this study

There has been little research in rural Africa on sustainable community-based interventions to reduce maternal and perinatal mortality, though this is changing. Government partners are involved in operational research in Ethiopia, Malawi, Mali, Mozambique, Tanzania and Uganda (Saving Newborn Lives, personal communication), and the Newhints trial in Ghana is looking at home visits by community-based volunteers [32]. Similarly, although there have been trials of behaviour change interventions for the promotion of exclusive breastfeeding in Mexico, Bangladesh and India, few published studies report similar interventions in rural Africa. Following on from the successes of community-based interventions in other settings in Asia and South America, we decided to test similar interventions in a rural African setting with high malaria and HIV. We believe this project will provide policy-relevant answers to key questions about strategies to improve mother and newborn health in Africa.

### Objectives

#### Goal

To improve the survival and health of mothers and infants in rural communities in Mchinji, Malawi.

#### Purpose

To test the effectiveness of two community-based health promotion interventions for improving mother and child health and reducing mortality.

#### Objectives

To test the impact of an intervention using community mobilisation through women's groups on:

a) Care practices and health care seeking behaviour for mothers and infants.

b) Maternal and infant morbidity.

c) Maternal, infant, neonatal and perinatal mortality.

To document and evaluate the process and costs for potential replicability and sustainability.

To test the impact of an intervention delivering health education through volunteer peer counsellors on:

a) Exclusive breastfeeding rates, other care practices and health care seeking behaviour.

b) Maternal and infant morbidity.

c) Infant mortality.

To document and evaluate the process and costs for potential replicability and sustainability.

## Methods/Design

### Study design

**Figure 1:**
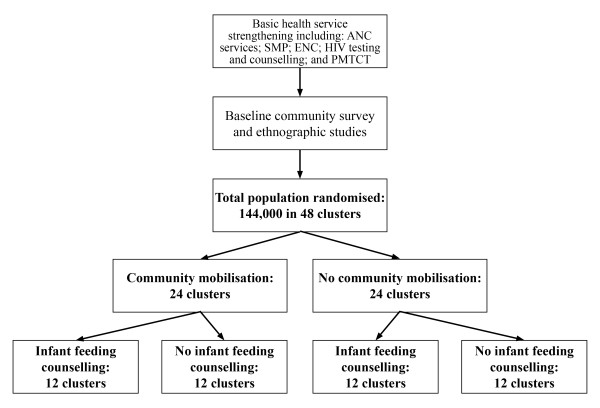
**Two-by-two factorial design**. Community-based women's group mobilisation intervention and home-based infant feeding and care counselling, with basic health service strengthening for both intervention and control areas

The intervention will be evaluated in a cluster-randomised controlled trial. A cluster design has been chosen because the allocation and loci of delivery of the interventions (community clusters) are groups rather than individuals. 48 rural clusters have been identified. 24 have been allocated to receive the women's group intervention and 24 will act as controls. Using a factorial design, each of these arms of the women's group trial was randomized a second time, 12 clusters to receive the volunteer counseling intervention and 12 to act as controls. An independent surveillance system for outcomes - births, morbidity, mortality, and care seeking - has been designed and implemented in all 48 clusters, covering a population of 144,000 (Figure 1).

### Primary research questions

Will community mobilisation through women's groups reduce perinatal, neonatal, infant and maternal mortality rates through changes in care practices and health-seeking behaviour?

Will volunteer infant feeding and care counselling for pregnant and breastfeeding mothers in their homes reduce infant mortality through changes in knowledge and practices regarding exclusive breastfeeding, family planning and other care practices and health-seeking behaviours?

### Hypotheses

Women's group activities will lead to: reductions in maternal, perinatal, neonatal and infant mortality, reductions in maternal and infant morbidity, and increases in recognition of high-risk symptoms, increased health-care seeking behaviour and changes in care-taker practices.

Infant feeding and care counselling sessions will lead to: reductions in infant mortality, reductions in maternal and infant morbidity, increases in exclusive breastfeeding rates in the first six months, increases in health-care seeking behaviour, and changes in care-taker practices.

### Study endpoints and outcomes

The study endpoints are shown in Table 1. The interventions will run for two years from the date when they are hypothesised to start having an effect, nine months after the start of the interventions, allowing for new pregnancies to have the maximum benefit of exposure to interventions. If a valuable effect is shown at the end of this period, the intervention models - refined on the basis of experience - will be implemented in the control areas.

**Table 1:** Study outcomes

	Women's groups	Volunteer infant feeding and care counsellors
Primary outcomes	▪ Maternal mortality ▪ Perinatal mortality ▪ Neonatal mortality ▪ Infant mortality	▪ Rates of exclusive breastfeeding (EBF) in the first six months ▪ Infant mortality

Secondary outcomes	▪ Recognition of high-risk symptoms and signs: failure to feed, breathlessness, floppiness. ▪ Changes in caretaker practices: hygiene behaviours, use of insecticide treated nets (ITNs), early and exclusive breastfeeding and decreased use of pre-lacteal feeds ▪ Changes in care-seeking behaviour: antenatal care (use of malaria prophylaxis in pregnancy, tetanus toxoid), delivery (facility-based, skilled attendant, use of safe delivery kit), uptake of PMTCT (VCT, nevirapine for mother and baby), postnatal care (check-ups for mother and baby, infant vaccinations), referral patterns. ▪ Maternal and infant morbidity (breast problems, fever, diarrhoea, etc.)	▪ Changes in caretaker practices: EBF (duration of EBF, time to first feed, use of pre-lacteals, time to weaning), management and treatment of breast problems, recognition of danger signs, birth preparedness (clean razor, clean plastic sheet, soap, thread), family planning (including use of condoms) ▪ Changes in care-seeking behaviour: uptake of PMTCT (awareness, VCT, nevirapine for mother and baby, expressing breastmilk, early cessation of breastfeeding), uptake of vaccination services (3 doses pentavalent and 4 doses polio by 6 months • Neonatal mortality • Maternal and infant morbidity (breast problems, fever, diarrhoea, growth etc.)

### Design and unit of randomisation

**Figure 2:**
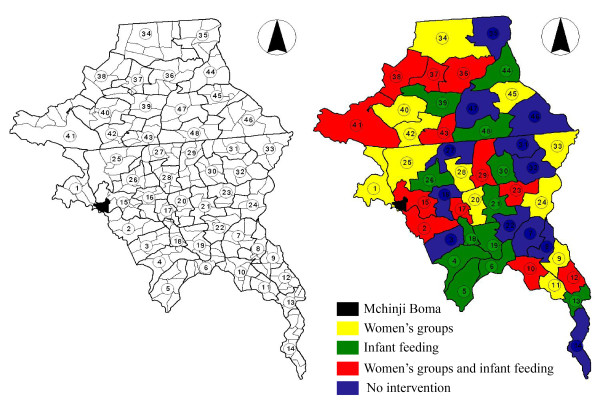
**a - Aggregation of census enumeration areas into 48 study 'zones', b - Random allocation of zones to four different combinations of intervention**.

**Figure 3:**
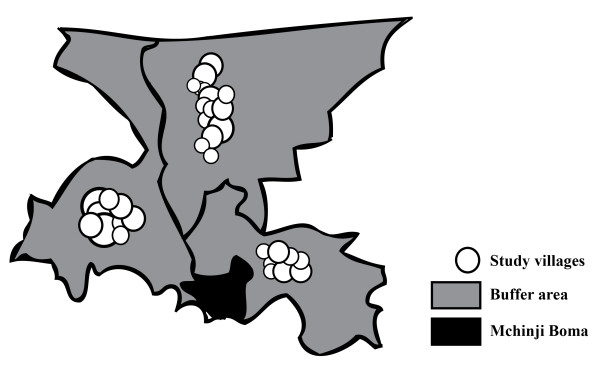
**Study villages and buffer areas in three clusters near Mchinji Boma**.

A cluster-randomised controlled trial, with a two-by-two factorial design, will be used to test the impact of two interventions on specific outcome indicators. 48 clusters or 'zones' were defined from the Enumeration Areas generated during the 1998 census (Figure 2a). Each zone contained a population of approximately 8000 in total. A population of around 3000 was selected from the centre of each zone, leaving a 'buffer area' around it to reduce contamination between neighbouring intervention and control areas (Figure 3).

### Setting

Mchinji district lies to the west of Lilongwe, in the Central Region of Malawi. It has international borders with Zambia and Mozambique, and a population of about 375,000 in 2004 [33], of whom about 80% live in rural areas and make a living through subsistence farming. The main ethnic group are the Chewa (90%), and the predominant religion is Christianity (97%). Comparison of some key socioeconomic and health indicators for Mchinji and Malawi are shown in Table 2.

**Table 2:** Socioeconomic and health indicators for Mchinji and Malawi

	Mchinji	Malawi
*Poverty*		

Human Development Index (out of 182 countries)	-	160
GNP per capita (US$)	-	690
Percent below $2 per day	-	90%

*Education*		

Female literacy (over 5 years of age)	46%	51%

Educational attainment - primary	60%	59%
- secondary	5%	8%

*Health*		

Access to improved water source	46%	45%
Access to sanitation	66%	53%
Total fertility rate (births per woman)	7.6	6.5
Crude birth rate (per 1,000 population)	55	50
Maternal mortality ratio (per 100,000 live births)	-	984
Infant mortality rate (per 1,000 live births)	-	76
Neonatal mortality rate (per 1,000 live births)	-	27

Sources: Malawi Population and Housing Census, 1998, Malawi DHS, 2004, World Bank 2006, State of the World's Newborns report 2001, UNDP 2009

In 2006 it was reported that 99% of women in Mchinji attended antenatal care at least once during their pregnancy, but only 58% delivered at a health facility [34]. Maternal and perinatal health care is provided by personnel from one district hospital (a first referral and secondary health facility), four rural community hospitals (one government and three mission hospitals), one maternity unit, six health centres that provide maternity care, two dispensaries and two private clinics that offer antenatal care. Quality is compromised by the severe shortage of personnel, low morale of the health providers, and irregular drug supplies. Traditional Birth Attendants are available in all localities.

The trial is being implemented by MaiMwana project, a Malawian trust established in 2003, as a collaboration between the Department of Paediatrics, Kamuzu Central Hospital, the Mchinji District Hospital and the UCL Centre for International Health and Development.

### Target group and eligibility criteria

The target population will be rural communities with the least access to health services, who might benefit most from community-based interventions to improve maternal and child health. The district administrative centre was excluded because it is more urbanised than the rest of the district and therefore not comparable to other clusters. The target group for participation in both of the interventions will be women of childbearing age (WCBA), between the ages of 15 and 49 years, and particularly pregnant mothers. Girls aged between 10 and 15 years will also be monitored and encouraged to participate in order to identify and support early teenage mothers. Older women will also be encouraged to attend as they influence decision-making around pregnancy, childbirth and child care and have valuable experiences to share.

All women aged 10 to 49 years (inclusive) who agree to take part, will be enrolled into the study, regardless of whether they are married or not. Women who have no possibility of conceiving during the study period will be enumerated but excluded from the final sample (for example women who have had hysterectomies or terminal family planning procedures). Participation in intervention activities will be voluntary, and women's groups will be free to establish their own membership criteria.

A cohort of 44,000 women aged between 10 and 49 years was defined during the baseline phase of the study, and each is visited monthly by study personnel. From the beginning of the study period we identified all pregnancies, births and deaths occurring within the cohort of WCBA, with follow-up until at least one year after delivery. The cohort members are listed in a master document to which new names can be added: the cohort is open and new participants may be enrolled during the study period if they move into the study area, or if they reach 15 years of age.

### Intervention activities

These are summarised in Table 3.

**Table 3:** Elements of the women's group and infant feeding and care counselling interventions

Women's groups	Volunteer infant feeding and care counsellors
*Specific objectives*	

Follow a participatory health education process to improve maternal and perinatal care	Make individual home visits to promote exclusive breastfeeding

*Elements of the interventions*	

The activities of Zonal Facilitators (ZFs) are the key to this intervention. Each facilitator will work within one Zone, covering an average population of 3,000. She will facilitate the activities of women's groups within the Zone as they address the issues of pregnancy, childbirth, newborn and infant health. Each women's group will move through a participatory planning cycle of assessment, sharing experiences, planning, action and reassessment, with the aim of improving essential maternal and newborn care.	The activities of Volunteer MaiMwana Counsellors (VMCs) will be the key to this intervention. Three VMCs will work within one Zone, covering an average population of 1,000 each. Each VMC will visit all pregnant mothers in her area 5 times - once before birth and four times after birth - to discuss the importance of exclusive breastfeeding, and to give support and advice on mother and child health. She will also help to identify any breastfeeding problems and refer them to a health facility.

#### Women's group intervention

**Figure 4:**
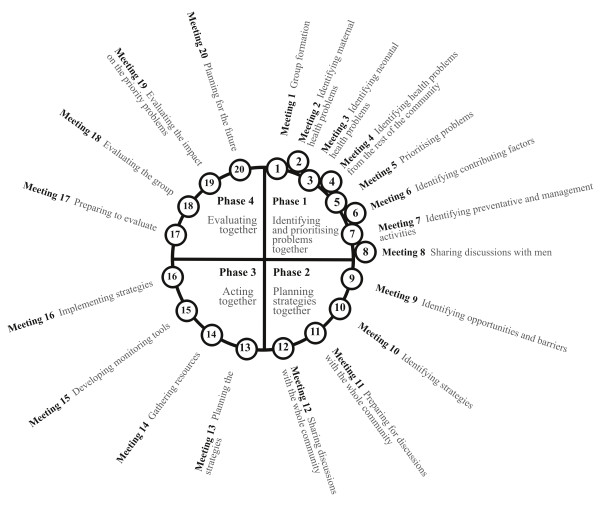
**Women's group cycle**.

The women's group intervention seeks to build the capacities of communities to take control of the mother and child health issues that affect them. The intervention is community-based in that it defines the community as the agent of change [35]. To achieve this, 24 local female facilitators, one per cluster, were recruited and trained. The facilitators formed between six and 12 groups in their clusters and invited all women of childbearing age to attend. They guide the groups of women through a four-phase community mobilisation action cycle developed to be appropriate, accessible and feasible for the Malawian context from similar models in Bolivia and Nepal (Figure 4) [36,37]. In the first phase, consisting of eight meetings, the groups identify and prioritise the mother and child health problems they feel are most important and discuss what may contribute to these problems and how they might be prevented and managed. In the last meeting of this phase they share their discussions with men in the community. In the second phase, consisting of four meetings, the groups plan locally feasible strategies to address the problems they have prioritised. In the last meeting of this phase they share their discussions with the whole community. In the third phase, consisting of four meetings, the membership of the groups expands to include all community members, including men, working together to implement the strategies that have been identified. In the fourth phase, four meetings, the groups evaluate what they have done, and plan for the future. The facilitators received minimal health training but use participatory rural appraisal tools and picture cards to facilitate discussions and enable groups to access their collective knowledge and capacities. With these capacities the groups take increasing control of the intervention over the course of the cycle. They do not receive any resources from MaiMwana Project, except the guidance of the facilitator, employed by MaiMwana project, and supported by four trained supervisors and a senior supervisor.

#### Volunteer infant feeding and care counselling intervention

The volunteer counselling intervention seeks to change the behaviour of individuals in relation to care and care-seeking for mothers and children. The intervention is community-based in that it defines the community as the target of change [35]. In particular, the intervention seeks to provide health education to raise the awareness, change the attitudes and build the self-efficacy of mothers in relation to exclusive breastfeeding. To achieve this, 72 female volunteers, between two and four per cluster, were recruited and trained to identify pregnant women in their area and visit them at home to provide counselling and support to exclusively breastfeed their infants and perform other key infant care practices. The volunteer counsellors visit these women at five key times in pregnancy and after birth (Table 4). The first visit is conducted in the third trimester, and the mother receives information regarding the importance of early and exclusive breastfeeding, PMTCT, birth preparedness and family planning. The second visit is conducted in the first week after birth (and where possible within the first three days), when the mother receives information on the importance of exclusive breastfeeding, vaccinations, warmth and hygiene and family planning. The mother also receives information about danger signs for herself and her child, advice about breast problems and support for good attachment and positioning. The third visit is conducted at one month after birth and covers the same issues as the previous visit, with the addition of information about weaning. The fourth visit occurs at three months after birth and covers the same issues as the third visit. The fifth visit is conducted at five months after birth and covers the same issues as the third and fourth visits with the addition of information about weaning foods. The volunteer counsellors received minimal health training but use a picture book to facilitate learning. The volunteer counsellors are supervised by 24 government Health Surveillance Assistants, and the intervention is coordinated by one supervisor employed by MaiMwana project.

**Table 4:** Volunteer infant feeding and care counselling intervention meeting guide

Visit 1	Visit 2	Visit 3	Visit 4	Visit 5
**Pregnancy**	**After birth**			

**3rd trimester**	**1st week**	**1 month**	**3 months**	**5 months**

*Introduction *Early BF *Exclusive BF *PMTCT *Birth-preparedness *Family planning and condoms	*Attachment & positioning *Exclusive BF *Vaccinations *Warmth *Hygiene *Danger signs *Family planning and condoms *Advice on BF problems	*Attachment & positioning *Exclusive BF *Vaccinations *Warmth *Hygiene *Danger signs *Discuss weaning at 6 m *Advice on BF problems	*Attachment & positioning *Exclusive BF *Vaccinations *Warmth *Hygiene *Danger signs *Discuss weaning at 6 m *Advice on BF problems	*Attachment & positioning *Exclusive BF *Vaccinations *Warmth *Hygiene *Danger signs *Discuss weaning at 6 m *Discuss weaning foods *Advice on BF problems

### Randomisation and allocation

The 48 study zones were randomly allocated to one of four groups; 12 zones receiving infant feeding counselling and facilitation, 12 zones receiving facilitation only, 12 zones receiving infant feeding counselling and no facilitation, and 12 zones receiving neither (Figure 1 and Figure 2b). All zones will benefit from the district-wide training and support for health services from the project's initiation.

Random number generation was done in STATA 7.0, and each of the 48 clusters was allocated to one of four possible combinations of interventions. In this way, each intervention was stratified according to the presence or absence of the other one, in order to balance any effects of one intervention on outcomes of interest in the other. SL and DO generated the random number sequence and allocated the clusters to intervention groups. All women aged between 10 and 49 years residing within the clusters were eligible for inclusion in the interventions and follow-up of maternal and child health outcomes. Records of a woman's migrations within and outside of the study area are recorded in order to allow for both 'intention to treat' and 'per protocol' analysis.

Due to the nature of the interventions, blinding of study participants to their allocation was not possible, though analysts and trial monitors will be blinded to the study allocation until the definitive analysis is performed. Data will be collected independently from intervention implementation, and no results will be fed back to inform the interventions.

### Sample size

The sample size for the cluster randomised controlled trial was arrived at by comparing statistical power for different estimates of population parameters. Parameters estimated included baseline mortality rates, the projected size of the reduction in maternal, neonatal and infant mortality and increase in exclusive breastfeeding rates due to the interventions, the number of births in each cluster over the trial period, the number of clusters in the intervention and control groups, the statistical power of the study, and the inter-cluster coefficient of variation. Realistic values of some of these parameters were difficult to estimate, as few population-level mortality data were available, especially at district and sub-district level. The sources of data and values used for these estimates and sample size calculations are summarised in Table 5. We aimed to achieve the smallest sample size that would allow adequate statistical power to detect an impact within a reasonable time-frame and would be logistically feasible to implement.

**Table 5:** Parameters used to estimate sample size, and the estimated size of reductions or increases that would be detectable

Parameter	Source of estimate	DHS parameter estimates (2006)
Number of clusters	Geopolitical subdivisions and logistical efficiency	48

Population per cluster	(Calculated)	3,000

Crude birth rate (per 1000 population)	National data from MDHS*	42

Time frame (years)	Funding period	2

Births per cluster within study period	(Calculated)	252

Inter-cluster coefficient of variation (k)	Hayes 1995	0.15-0.3

Statistical power of the study	Probability of Type I error Probability of Type II error	0.05 0.2

Neonatal mortality rate (per 1000 live births)	National data from MDHS*	27

Size of reduction detectable	(Calculated)	31-36%

Maternal mortality ratio (per 100,000 live births)	National data from MDHS*	984

Size of reduction detectable	(Calculated)	47-50%

Infant mortality rate (per 1000 live births)	National data from MDHS*	76

Size of reduction detectable	(Calculated)	21-28%

Exclusive breastfeeding (%)	National data from MDHS*	27.5%

Size of increase detectable	(Calculated)	16-30%

* Malawi DHS data used is the national estimate, as data were not disaggregated for Mchinji District

Initial sample size estimates were made using national estimates of crude birth rates and mortality rates from the Malawi DHS for 2000 [38], subsequently revised after the results for the 2004 DHS survey were released [2]. Estimates were made with 80% power, a two-sided 5% significance level and an inter-cluster coefficient of variation (k) of between 0.15 and 0.30, using the methodology laid out by Hayes *et al *[39]. An estimate of k = 0.25 came from work on interventions to reduce HIV incidence rates [40], and later from work in Makwanpur, Nepal [27]. We assumed that variability in neonatal, infant and maternal mortality between clusters would be less in rural Malawi, due to fairly uniform exposure to risk factors such as poor hygiene and poor quality or absent delivery care and malaria. In addition, we reduced potential heterogeneity by excluding the main district administrative centre, as it was felt to be socio-economically and demographically different from rural villages. In recognition of the lack of certainty for this estimate, sample sizes were calculated for a range of values of k.

### Interactions between the women's group and volunteer infant feeding and care counselling intervention

Through the two-by-two design, it will be possible to assess the interaction between the two interventions, though the study is not powered to evaluate the combined impact of the interventions on mortality compared to single interventions alone, as this would have required an unfeasibly large sample. We will explore qualitatively and quantitatively whether or not there is a synergistic relationship between the two interventions, resulting in an effect greater in magnitude than either intervention alone. We might expect that women in an enabling environment (provided by women's groups) would find it easier to discuss health issues and make health-seeking decisions for themselves and their babies than women in control areas where social barriers to care-seeking have not been addressed. More specifically, women in areas receiving both interventions might be more likely to use the Volunteer MaiMwana Counsellor and recognise the importance of her advice. More of these women may have decided to use PMTCT services than those not receiving facilitation, and will therefore be in a better position to make informed choices about infant feeding and family planning based on knowledge of their HIV status. Conversely, individual visits made by volunteers to women in their homes may serve to reinforce messages and issues arising from women's group discussions.

### Data collection

Data will be collected in two phases; baseline data during intervention development, and prospective data during intervention implementation. The baseline phase was completed in 2004. Formative qualitative data were collected through semi-structured interviews and focus groups. These data were collected with the specific purpose of: a) exploring current care practices to help to develop the structured questionnaires and interviews; and b) exploring the aims, setting, target population, methods and resources of the interventions to assist in their development. During the baseline data collection phase, the 692 study villages were mapped, each household enumerated, and a census was done in order to collect basic socioeconomic and demographic data about household members. A list of all female residents aged between 10 and 49 years was produced.

Prospective data relevant to the evaluation of the two interventions will be collected in three ways - impact, process and economic.

#### Impact evaluation

To assess the impact of the interventions on mortality, all WCBA will be visited monthly to identify pregnancies and births, as well as neonatal, infant and maternal deaths. WCBA will be visited by trained enumerators once per month, and events recorded in a register holding an up-to-date list of all women in the cluster (generated from the baseline survey, plus new residents). One enumerator will visit all WCBA in one cluster. All deaths of infants and women will be followed up by a supervisor who will verify the death and conduct a verbal autopsy interview between two and six weeks after the death. This interview will help to elicit the causes and contributing factors of the deaths [41]. There are five supervisors, each based at a nodal office.

To assess the impact of the interventions on morbidity, care practices and behaviours, women who are identified as pregnant will be followed up until 6 months after birth by trained interviewers. Interviewers will administer one-month and six-month post-partum interviews to collect detailed information about demographic characteristics, maternity history, health-seeking behaviours, care behaviours and maternal and infant morbidity.

To assess the impact of the interventions on growth and nutrition, women and infants will be visited by Health Surveillance Assistants (HSAs) at one-month and seven-months to collect anthropometric and biodata. Infant weight and height and mother's mid-upper arm circumference will be measured, and blood spots taken from infant heel pricks will be tested for haemoglobin levels using a Haemocue machine. Six HSAs have been recruited and have received extensive training according to Malawi government guidelines in theoretical and practical aspects of blood spot collection by the Community Health Sciences Unit (CHSU) technicians. The HSAs will also be supervised by CHSU technicians quarterly and attend full week-long six-monthly refresher trainings.

#### Process evaluation

An integrated process evaluation will collect data on the key factors that may mediate the impact of the interventions in order to understand why they work or fail to work, and how different contextual factors influence their success. This will include collecting information on the context in which the interventions are being implemented, the mechanism through which the interventions are working, and the proximal outcomes of the interventions. On the basis of this information, hypotheses will be developed and, where possible, tested to explain and better understand the impact of the interventions. The data will be collected, using predominantly qualitative methods such as key informant interviews, focus group discussions and observations. A range of different respondents will be consulted, including members of intervention and control communities, members of women's groups and women who have been counselled, key community, district and national informants, MaiMwana staff, and health facility staff.

#### Economic evaluation

All inputs will be audited and cost-effectiveness analysis will be carried out in order to assess the replicability and scalability of the interventions, and the potential for them to be adopted as larger scale public health interventions in Malawi. Full costs will include start-up and running costs for both interventions, and will be collected through the project accounting systems. In addition to financial costs, economic costs will be estimated, valuing resources at their "opportunity cost", or value in their best alternative use (including time, resource use and donated items) [42]. We will estimate the cost per maternal, infant, neonatal and perinatal death averted and the cost per newborn infant exclusively breastfed. We will collect information on the costs associated with monitoring and evaluation, although research costs will not be included in the analysis. Appropriate sensitivity analyses will be used to explore the implication of uncertainty of assumptions. The cost of scaling up the interventions at national level will be estimated in order to explore cost-saving opportunities and to investigate issues of generalisability beyond the trial context and beyond domestic boundaries.

### Data management

All quantitative data collected will be delivered to the main office for data entry in a relational database management system in Microsoft Access run on a dedicated server and workstations. Each WCBA will be given a unique ID number generated from the cluster, village and household she comes from. All quantitative data from the mortality surveillance, morbidity, care practice and behaviour questionnaires and process evaluation will be linked to the WCBAs through this unique ID. After checking and entry, all questionnaires will be archived in a locked room for future reference.

Qualitative information will be audio recorded after receiving verbal consent from respondents. Data in Chichewa will be translated into English by a bilingual speaker. To ensure conceptual, grammatical, and syntactical equivalence, translations will be reviewed collaboratively by the researcher who obtained the data and the translator (both bilingual Chichewa-English speakers), and the lead researcher (English speaker). This team will make decisions about the best terms to use. All data will subsequently be transcribed and imported into a database in MAXqda 2 (VERBI Software version 2).

### Quality control

One enumerator per cluster will identify births and deaths, and each event will be cross-checked by one interviewer. Supervisors will make regular field visits to check the quality of work done by enumerators and interviewers and observe some interviews. Each supervisor is responsible for between six and ten clusters. Interviewers will meet with enumerators weekly in order to check on their work and receive updates on births and deaths in their area. Supervisors will meet with interviewers and enumerators fortnightly to check on their work, discuss problems and provide quality control feedback. A sample of 200 one-month and six-month interviews has been selected to be independently re-done by the supervisor, in order to be able to estimate recall and interviewer error rates.

Quantitative data will be checked in three stages. The first check will be performed after completion of the questionnaire, by the MEO *in full *and a nodal data checker (NDC) based at one of the five nodal offices. The second check will be done by a team of two data checkers (DC) based at the main office. The last stage of data checking will be done at the point of data entry by the four data entry clerks (DEC). Further checks will be carried out internally within the electronic data-handling environment. Data entered into the study databases will be regularly reviewed for inconsistencies and missing information. Lists of women interviewed and key fields to be verified will be produced, such as ID numbers, dates of birth, and reported pregnancies, births or deaths that have received no further follow-up.

### Dealing with loss to follow-up

Minimising loss to follow-up is an important aspect of trial conduct. Certain features of this location and population dynamic need special attention for outcome tracking: a) Residents of Mchinji move seasonally to maximise their access to fertile land during the farming season, both within the district and across international borders into Zambia and Mozambique; b) Residents of Mchinji may go home for delivery and some time after birth (to other villages within Mchinji or to other districts); c) Non-residents may come into Mchinji from other districts for delivery and some time after birth; d) Families may move after a woman's death, making it difficult to find respondents who know the details of what happened; e) High population turnover in trading centres and commercial farm estates; f) Women being busy working in their gardens, or at community gatherings such as funerals and chieftainship ceremonies, making them unavailable for interview; g) Weather conditions making roads impassable and conducting interviews difficult.

For residents or respondents who are temporarily unavailable, the main strategy is to keep following up until an outcome is ascertained. All women who have ever lived in the study areas are maintained in the database, and appear every month in the register. Any events (such as pregnancy or birth) that are reported but no further details are known, are selected and lists produced to remind field-workers of the need for follow-up. In most cases this causes delays in getting complete data, though basic data for estimating mortality rates are still available. In recognition of the fact that certain data collected after a long delay will no longer be valid, sections in the questionnaire (such as infant feeding recall) are skipped.

For residents or respondents who are permanently unavailable, basic information about dates and timings of events is sought from other community members such as friends or neighbours.

### Reducing contamination

Contamination may occur when people from one cluster have contact with people from another. In the rural villages of Mchinji, there are many opportunities for social mixing. Friends, relatives or neighbours may mix socially, or contact may be made through travel or migration between intervention and control clusters. There might be direct participation of residents from control areas in WG or VMC activities, or more likely, informal discussion of ideas from WG or VMC activities - control area residents may gain some benefit from hearing health messages received by intervention participants. The usual effect of this kind of contamination would be 'dilution' of the differences between treatment arms [43].

In order to reduce the possibility of contamination, we opted to use clusters of villages rather than individual villages as the unit of randomisation, thus reducing rates of travel across cluster boundaries [43]. Furthermore, each zone had a defined 'buffer area' around the perimeter (figure 2). A population of 3000 in each zone was required to achieve the desired sample size, but rather than selecting villages at random from each zone, only villages at the centre of the zonal area were eligible for inclusion in surveillance and intervention activities. This reduced the possibility of communication between neighbouring study villages in intervention and control areas.

Women's group facilitators and volunteer counsellors are residents of the zone in which they work. This reduces the possibility that they might transfer intervention benefits to neighbouring communities. For the WG trial, whilst health messages are discussed in group meetings, it is unlikely that neighbouring control communities would spontaneously mobilise themselves without the presence of a facilitator. So the purported benefits of community empowerment and social capital are unlikely to spread beyond intervention areas.

### Interim analyses and stopping rules

An independent Data Safety and Monitoring Board (DSMB) will meet several times according to the DAMOCLES statement, [44] to review progress and advise on the conduct of the trial. They will assess compliance with the protocol, data quality and completeness, recruitment figures, sample size assumptions and ethical considerations. The meetings will not include analysis of outcome data by intervention allocation until completion of the trial. Baseline data will be reviewed to evaluate how well balanced the clusters were after randomisation and suggest any adjustments that may need to be made. We do not foresee any adverse effects of community mobilisation or peer counselling, so we do not intend to apply stopping rules.

### Analysis plan

Data from the trial will be analysed by intention to treat at both cluster and participant levels. Participants will be assigned to the cluster they were resident in at the time the trial began, even if they move to another cluster or out of the study area during the trial period. Primary and secondary outcomes will be compared between intervention and control groups with random effects logistic regression models, taking account of clustering. Potential confounding factors will be included in the model, and measures of baseline mortality will be included in order to adjust for any imbalances in randomisation at the outset. Cluster-level data will be compared using modified t-tests. All estimates will be presented with 95% confidence intervals.

Data collected from the project both during the baseline phase and the intervention phase mean that we will be able to look at overall changes in outcome indicators over time. Factors other than the study interventions may influence health indicators seen, such as other mother and child health programs in the area, or positive reporting and the Hawthorne effect [45]. If mother and child health programs are only active within some of the cluster areas then these can be examined separately if necessary.

Analysis of qualitative data will involve iteratively developing a system of codes and memos jointly by the lead researcher and the researcher who collected the data. This method will seek to develop an analytical framework based on the data by coding and memoing pertinent excerpts that illustrate emerging themes. Subsequently, in an iterative process, the researchers will refine their analysis ensuring that the themes that are built up are cross-checked with other data firstly within and then between transcripts so that the validity of emerging explanations is tested and improved. The qualitative data will also be used to interpret and contextualise the quantitative data collected. MAXqda 2 software will be used to facilitate this analysis.

### Ethical issues

#### Approvals

Ethical permission for this study was granted by the Malawi National Health Sciences Research Committee in January 2003 (Ref: MED/4/36/I/167) and the ethics committee of the UCL Institute of Child Health and Great Ormond Street Hospital. It is registered with ISRCTN06477126.

#### Community consultation

Verbal and written consent was received from community leaders after full consultation and discussions. The regional, district and village leaders, and local health and development professionals will have ongoing access to the research programme and will be the first to be briefed on study findings and outcomes through written and verbal reports.

#### Individual consent

Before each instance of data collection, the process and advantages and disadvantages of taking part will be explained to all participants. Verbal consent will be obtained, and participants informed that they can stop taking part at any time. Participation in intervention activities is voluntary, and women may choose to start or stop as they wish.

#### Benefits to the control communities

The study is designed to test the community effectiveness of two community-level initiatives to reduce maternal, infant, neonatal and perinatal mortality in rural Africa. Encouraging community action for maternal and newborn care alone will not grant success. For health promotion interventions to work, the supply side of health care services must reach a minimum standard. The study team considers it unethical to strengthen services only in intervention and not control areas. Control communities will benefit from low-cost improvements in equipment, supplies and training at all primary level facilities in the district in intervention and control areas.

#### Treatment of illness in participating communities

When the study workers identify minor or chronic illness in mothers or infants in either intervention or control areas they will encourage referral to the appropriate health facility.

#### Confidentiality of information

All information will remain confidential. Access to information will be limited to interviewers and their supervisors at sites of collection, to auditors and data feeders at the collation point and thence to the senior data management officers and principal investigators. No analyses or reports will include the names of participants.

### Sustainability and scalability

A key objective is to take lessons learned quickly to scale in other districts, through our partners. The Ministry of Health in Malawi will be involved throughout the implementation of this project. At a local level we will work closely with the district development committees, local community representatives and locally active NGOs. The Mchinji District Health Office will play a key collaborative role. We will also work closely with UNICEF, Malawi and the National AIDS Commission, to implement PMTCT activities.

Community-based team members have been recruited locally and carry out their activities in their home areas. We will attempt to maintain intervention costs to a minimum so that they can be adopted by the Ministry of Health.

Control areas will be the first beneficiaries of scale-up if either of the interventions prove to have a positive impact.

## Competing interests

The authors declare that they have no competing interests.

## Authors' contributions

CM is the project coordinator, contributed to the design of the study, will be responsible for the management of the trial, and will participate in the analysis and interpretation of data. SL contributed to the design of the study, will coordinate the data collection and lead the analysis and wrote the first draft of the study protocol. PK, MLN, DO and AC contributed to the design of the study and will participate in the analysis and interpretation. TP, MR, HC, FM, AM contributed to the design of the interventions, will be responsible for their implementation, and will participate in the analysis and interpretation of data. SV contributed to the design of the infant feeding interventions and health service strengthening. CM is the director and TP the project manager of MaiMwana, contributed to the design of the study, and will have overall responsibility for the trial in Malawi. AC is the director of the UCL Centre for International Health and Development, contributed to the design of the study, and will have overall responsibility for UK partner contributions. MR and FM contributed to the design of the interventions and the process evaluation, and will participate in the analysis and interpretation of data. AC, SL, CM, PK, MLN, MR, SV and DO obtained grant funding. All authors contributed to critique and modification of the manuscript.
